# Intranasal Oxytocin Increases Head Motion During Functional MRI Scanning

**DOI:** 10.21203/rs.3.rs-3912105/v1

**Published:** 2024-02-07

**Authors:** Sydney Houlton, Jatin G. Vaidya, Patrick Breheny, Lane Strathearn

**Affiliations:** 1 Department of Pediatrics, University of Iowa, Iowa City Iowa, USA.; 2 Department of Psychiatry, University of Iowa, Iowa City, Iowa, USA.; 3 Department of Biostatistics, University of Iowa, Iowa City, Iowa, USA.

**Keywords:** functional MRI, oxytocin, head motion, framewise displacement, addiction

## Abstract

Oxytocin is a neuropeptide associated with prosocial behaviors, such as parent-child bonding, eye contact, and sexual activity. Intranasally-administered oxytocin has been widely used to study its effects on the brain using functional magnetic resonance imaging. Head motion is a significant confounding variable which was assessed as part of a double blind, placebo-controlled crossover study. Twenty-four mothers with drug addiction problems were initially recruited, along with 22 healthy control mothers, to test whether intranasal oxytocin enhances functional brain responses to images of their own versus unknown infant faces. Significant differences in head motion between oxytocin/placebo conditions and addiction/control groups were discovered. Administration of intranasal oxytocin was associated with more frequent counts of head motion exceeding 3 mm of framewise displacement, independent of group status (*z*=2.89, *p*=0.004). This effect was seen more strongly in the control group (*z*=2.30, *p*=0.02) than the addiction group (*z*=1.77, *p*=0.08). The addiction group was more likely to show increased head motion, independent of oxytocin or placebo condition (*z*=2.21, *p*=0.03). When examining the mean head motion across all time points, as opposed to the count of large movements, oxytocin’s effect was limited to the addiction group (*z*=2.58, *p*=0.01), with a significant group by condition interaction effect observed. Intranasally-administered oxytocin may therefore have a confounding effect on functional MRI scanning results via its independent effect on head motion. These findings should be examined and replicated in other clinical populations.

## INTRODUCTION

Oxytocin is a neuropeptide synthesized in the paraventricular nucleus and the supraoptic nucleus of the hypothalamus (Born et al., 2002). In addition to stimulating the milk letdown reflex and uterine contractions, oxytocin is associated with prosocial behaviors such as hugging, sex, eye contact, and bonding (Bethlehem et al., 2013; Peltola et al., 2018). Oxytocin has been studied extensively in humans using intranasal delivery to examine its behavioral effects and the changes in neural activation. The central effects of intranasal oxytocin have been validated by measuring the oxytocin concentration in cerebrospinal fluid (CSF) after administration (Born et al., 2002). The effects of oxytocin on behavior and brain response have been studied rigorously using randomized double-blind placebo-controlled trials (Alaerts et al., 2022; Ebner et al., 2016; Lan et al., 2023). Intranasal oxytocin has also been explored as a potential pharmacological treatment of psychiatric conditions involving social and emotional deficits, such as autism, anxiety, and depression (Born et al., 2002).

A significant limitation when using functional magnetic resonance imaging (fMRI) is head motion. Movements as subtle as breathing and involuntary twitching can affect the quality of the data collected in the scanner (Power et al., 2012). Head motion can distort the images or even render them unusable for data processing. An established measure used to quantify movement in the scanner is framewise displacement (FD), which is an index of head movement in six translational and rotational axes from volume to volume. The change of neural activation can be measured using DVARS, quantified as a frame-by-frame rate of change in blood-oxygen level dependent (BOLD) signal. In addition to distorting the images, motion in the scanner can alter the neural activation patterns observed, with FD being positively correlated with DVARS measures (Afyouni & Nichols, 2018; Power et al., 2012).

The purpose of this study was to determine whether head motion was associated with group or condition differences, as part of a randomized double-blind placebo-controlled crossover study of intranasal oxytocin in mothers with addictions versus control mothers.

## METHODS

### Design.

During a behavioral testing visit, infant faces and cries were collected for use during the scanning sessions (Kim et al., 2017) and participants self-reported on a range of sociodemographic variables, breastfeeding status, and completed the Beck Depression Inventory and the Beck Anxiety Inventory. Each participant then attended two identical fMRI scanning visits, distinguished only by administration of either oxytocin or placebo sprays in a randomized order. All study procedures were approved by the Institutional Review Board of the University of Iowa.

### Groups.

46 mothers aged between 18 and 40 years, with their 4–12-month-old infants, were recruited into the study and completed the first scanning visit. Thirty-five mothers completed the second scanning visit. Twenty-four of these mothers had been recruited into the addiction group from the University of Iowa Hospitals and Clinics (UIHC) and the Heart of Iowa residential substance use disorder treatment program, meeting DSM criteria for a substance-use disorder. Twenty-two mothers were recruited into the control group from UIHC and the surrounding community.

### Conditions.

Each mother self-administered either 24 international units (IU) of oxytocin (6 IU per spray, 2 sprays per alternating nostril) or a water-based placebo spray, approximately 50 minutes prior to the scanning sessions. Participants and investigators were blind to the composition of the nasal sprays. The spray order was counterbalanced, with half of the participants receiving oxytocin prior to the first scanning session and half receiving the placebo spray first.

### fMRI Paradigm.

While in the scanner, mothers viewed four different types of infant faces (their own infant’s happy face, an unknown infant’s happy face, their own infant’s sad face, and an unknown infant’s sad face), and listened to 2 sec infant cries (from their own or an unknown infant), and were asked to press a button on seeing and hearing a bell (Kim et al., 2017). All images/audio clips appeared in a pseudorandom sequence with a stimulus duration of 2 seconds and a random interstimulus interval of 2–11 seconds. The same stimuli were presented in a different order for the 4 sequential scanning runs.

### Data analysis.

Addiction and control groups were compared on several sociodemographic and mental health variables using Fisher Exact Test. A *p*-value<0.05 was considered statistically significant.

Standard fMRI preprocessing steps were performed using *fMRIPrep* for each participant (Esteban et al., 2019) (Supplementary Information). FD was computed using two formulations following Power et al. (2014) (absolute sum of relative motions) and Jenkinson et al. (2002) (relative root mean square displacement between affines). Not all participants completed both scanning sessions or all 4 runs, so FD and DVARS were calculated from a total of 81 functional runs (18 Addiction-Oxytocin, 21 Addiction-Placebo, 21 Control-Oxytocin, 21 Control-Placebo), using the implementations in *Nipype,* following the definitions by Power et al. (2014).

Mixed models were fit using a random effect for subject to account for repeated measurements: negative binomial mixed models were used to analyze the incidence of large displacements (FD ≥3 mm), and linear mixed models were used to analyze the mean FD, while accounting for potential overdispersion. Run number and group by condition interactions were also included in the model. Order of nasal spray presentation (oxytocin or placebo administration during first scan) did not predict outcomes and was therefore excluded from the final model. All analyses were conducted using R version 4.2.1, and mixed models were fit using lme4 (R Core Team, 2022). Scripts and codes used to generate results are stored at: https://github.com/pbreheny/oxytocin-addiction-imaging.

## RESULTS

Mothers in the addiction group were more likely to be disadvantaged in multiple domains, reporting low income, unmarried status, and lower education levels. They were also less likely to be breastfeeding their infant (Supplementary Table 1).

**Figure 1.**
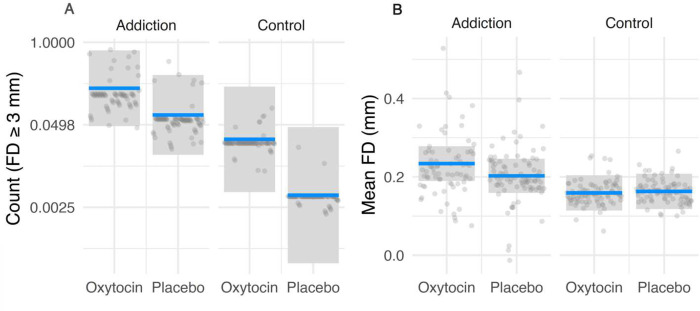
Intranasal oxytocin administration, compared to placebo, is associated with increased head motion during function MRI scanning. The left panel (**A**) shows the differences in the incidence of FD ≥ 3mm between group and condition. The right panel (**B**) shows the differences between group and condition in the mean FD within each functional run. The blue line and grey box represent the maximum likelihood estimate (MLE) and 95% confidence interval, respectively, for the corresponding mixed model response, conditional on group, condition, and run. The grey dots represent the partial residuals of the model fit. In panel B, a single outlying point in the Addiction-Oxytocin group was omitted, to enhance clarity.

Contrary to the hypothesis, administration of intranasal oxytocin was associated with an increase in head motion, independent of group status (*z*=2.89, *p*=0.004), using a 3mm threshold of framewise displacement. This effect was seen more strongly in the control group (*z*=2.30, *p*=0.02) than the addiction group (*z*=1.77, *p*=0.08). As hypothesized, the addiction group was more likely to show increased head motion, independent of oxytocin or placebo condition (*z*=2.21, *p*=0.03). No significant group by condition interaction was observed (Table 1A, Figure 1A).

There are no significant differences in condition for lower thresholds of head motion (FD ≥ 0.5 mm or 1 mm), although there were significantly more FD counts exceeding 0.5 mm in the addiction group, both overall (*z*=2.15, *p*=0.03) and with oxytocin condition specifically (*z*=2.26, *p*=0.02) (Supplementary Table 2).

When examining the mean head motion (FD) across all time points, oxytocin’s effect was limited to the addiction group (*z*=2.58, *p*=0.01), with a significant group by condition interaction effect seen (*z*=2.18, *p*=0.03). Likewise, disadvantaged mothers with addiction problems only showed significantly increased head motion in the oxytocin condition (*z*=2.34, *p*=0.02) (Table 1B, Figure 1B). Head motion also increased linearly across all 4 scanning runs, independent of group and condition (*z*=2.62, *p*=0.01).

When looking at the full dataset, containing all timepoints, FD and DVARS had a Pearson’s correlation of R = 0.33 (*p*<0.0001), suggesting a strong association between head motion and BOLD activation.

## DISCUSSION

In this randomized, double-blind, placebo-controlled crossover study, intranasal oxytocin administration was associated with larger head movements (FD ≥ 3 mm), independent of group status. In addition, head motion was associated with change in fMRI BOLD signal, as measured by the significant correlation between FD and DVARS. Thus, head motion, most commonly measured using FD, may be an important variable to consider in all fMRI studies using intranasal oxytocin. This is especially due to its association with distorted image quality and BOLD signal, as measured by DVARS.

Oxytocin’s association with head motion may be at least partially explained by its modulatory effects on dopamine via nigrostriatal pathways in the brain, leading to differences in locomotor control (Bethlehem et al., 2013). One preclinical study of male rodents suggested that oxytocin may affect motion via connections with the dorsal striatum (Angioni et al., 2016).

As hypothesized, mothers with addiction problems were also more likely to display higher levels of head motion, at both low and high motion threshold levels (FD ≥ 0.5 mm and ≥ 3 mm) (Table 1 and Supplementary Table 2). Nevertheless, it is unclear whether this was related to the addiction status per se, or other co-existing measures of more general disadvantage, including low income, unmarried status, and/or lower education levels (Supplemental Table 1). If this is the case, these results may be applicable to a much broad research population.

## CONCLUSION

Intranasal oxytocin is frequently used in fMRI studies to test its effect on social or attachment-related brain responses (Zhang et al., 2021). Although replication in additional patient populations is needed, these results suggest that oxytocin may have an independent effect on head motion and BOLD signal response in the scanner. Thus, intranasal oxytocin may alter brain activation patterns via non-pharmacological means, and have an important confounding effect on functional neuroimaging results.

## Figures and Tables

**Table 1:** Mixed models examining how *Condition* (intranasal oxytocin versus placebo administration), *Group* (addiction versus control participants), and *Run* (fMRI run sequence) predict differences in head motion (framewise displacement, FD) as measured by **A**) count of incidents in which FD≥ 3mm, and **B**) mean FD.

	A. Count: ≥3mm FD				B. Mean FD			

	Estimate	Std. Error	*z* value	*p* value	Estimate	Std. Error	*t* value	*p* value

**Condition Contrast: Oxytocin vs. Placebo**								
Both Groups	1.50	0.52	2.89	0.004	0.01	0.008	1.70	0.09
Addiction	0.97	0.55	1.77	0.08	0.03	0.01	2.58	0.01
Control	2.03	0.88	2.30	0.02	−0.004	0.01	−0.36	0.72

**Group Contrast: Addiction vs. Control**								
Both Conditions	2.39	1.08	2.21	0.03	0.06	0.03	1.86	0.07
Oxytocin	1.86	1.07	1.74	0.08	0.07	0.03	2.34	0.02
Placebo	2.92	1.32	2.21	0.03	0.04	0.03	1.25	0.22

**Run**	0.02	0.18	1.22	0.22	0.01	0.00	2.62	0.01

**Group x Condition Interaction**	−1.06	1.04	−1.02	0.31	0.04	0.02	2.18	0.03

## Data Availability

Anonymized head motion analysis data are available at: https://github.com/pbreheny/oxytocin-addiction-imaging.
